# Automated Axon Counting in Rodent Optic Nerve Sections with AxonJ

**DOI:** 10.1038/srep26559

**Published:** 2016-05-26

**Authors:** Kasra Zarei, Todd E. Scheetz, Mark Christopher, Kathy Miller, Adam Hedberg-Buenz, Anamika Tandon, Michael G. Anderson, John H. Fingert, Michael David Abràmoff

**Affiliations:** 1 Stephen A. Wynn Institute for Vision Research, University of Iowa, Iowa City, IA 52242, USA; 2 Department of Biomedical Engineering, University of Iowa, Iowa City, IA 52242, USA; 3 Department of Ophthalmology and Visual Sciences, University of Iowa Hospitals and Clinics, 200 Hawkins Drive, Iowa City, IA 52242, USA; 4 Department of Veterans Affairs, Iowa City VA Medical Center, 601 Highway 6 West, Iowa City, IA 55242, USA; 5 Department of Molecular Physiology and Biophysics, University of Iowa, Iowa City, IA 52242, USA; 6 Department of Electrical and Computer Engineering, University of Iowa, Iowa City, IA 52242, USA

## Abstract

We have developed a publicly available tool, AxonJ, which quantifies the axons in optic nerve sections of rodents stained with paraphenylenediamine (PPD). In this study, we compare AxonJ’s performance to human experts on 100x and 40x images of optic nerve sections obtained from multiple strains of mice, including mice with defects relevant to glaucoma. AxonJ produced reliable axon counts with high sensitivity of 0.959 and high precision of 0.907, high repeatability of 0.95 when compared to a gold-standard of manual assessments and high correlation of 0.882 to the glaucoma damage staging of a previously published dataset. AxonJ allows analyses that are quantitative, consistent, fully-automated, parameter-free, and rapid on whole optic nerve sections at 40x. As a freely available ImageJ plugin that requires no highly specialized equipment to utilize, AxonJ represents a powerful new community resource augmenting studies of the optic nerve using mice.

Glaucoma is a disease of the optic nerve and a leading a cause of irreversible vision loss that affects over 60 million people worldwide. Glaucoma is characterized by apoptosis of retinal ganglion cells (RGCs), resulting in a loss of their axons comprising the optic nerve and corresponding visual field loss^1^,^2^. Precise mechanisms of ganglion cell and axon loss in glaucoma remain incompletely understood. One approach to study glaucoma has been through utilization of mouse models^3^,^4^, which provide the opportunity for detailed studies of disease mechanism and testing potential treatments. The gold-standard assay for describing glaucomatous damage in mice is to quantify the number of axons in the optic nerve, which are typically manually counted by human experts after sacrifice, preservation and staining with paraphenylenediamine (PPD)^5^,^6^,^7^. However, a significant challenge to axon quantification is related to natural variability in their number; different strains of mice can have a 2 fold difference in naturally occurring axon number, and even within genetically identical mice, the coefficient of variation for axon number is ~3.6%^8^. Use of different counting methodologies by different research groups adds additional variability, as illustrated by studies of axon number in adult C57BL/6J mice, which are typically reported as 45,000 to 55,000^8^,^9^,^10^,^11^, but have ranged from <25,000^12^ to >70,000^13^. Manual counting is also time-consuming, so it is typically applied to 10% or less of any sample, and prone to intra- and inter-observer variability. In sum, there are numerous challenges to accurately quantifying optic nerve axon numbers in mice and considerable opportunities for methodological improvements.

Here, we report development of a fully automated algorithm to count all axons in PPD stained optic nerve cross sections, as well as measure axon density and axon size distribution. To achieve this, we modified our existing image-analysis algorithms, previously developed for general object background separation and segmentation^14^,^15^,^16^,^17^, for use in axon counting as AxonJ, a plugin for ImageJ^18^. AxonJ is freely available to the wider research community through the ImageJ plugin repository (http://imagej.nih.gov/ij/plugins/axonj/)]^19^. The purpose of this study is to describe the algorithm, validate its performance on full optic nerve cross-sections from multiple strains of mice and compare its performance to human experts.

## Results

### AxonJ Image Analysis Algorithm

**Figure 1:**
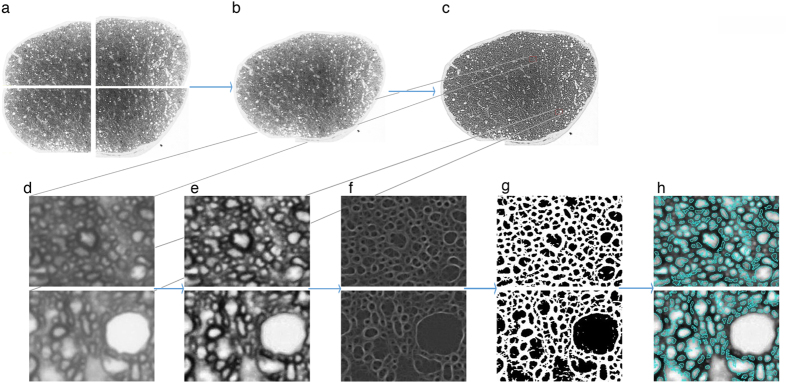
AxonJ flowchart of algorithmic steps. (**a**) Raw 40x magnification images (single mouse in image set T0a) covering the whole optic nerve section (**b**) registration results in single whole optic nerve image (**c**) whole optic nerve after histogram equalization (**d**) Details of 40x magnification images (**e**) after local histogram equalization (**f**) after Hessian transformat g) after thresholding h) connected regions.

**Figure 2:**
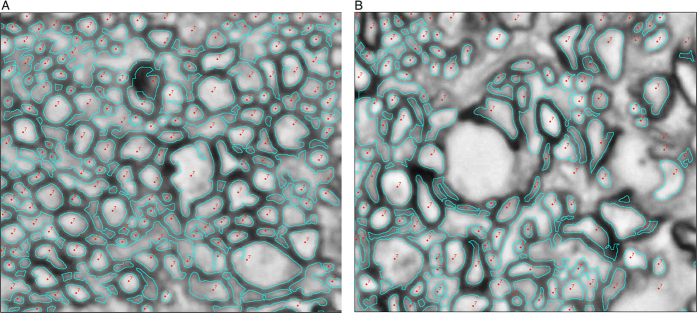
Examples of expert and AxonJ identified axons in PPD-stained 100x optic nerve sections (image set A, Table 1). Axon detections of expert (red point labels) and AxonJ (turquoise outlines), on a cropped 100x sample from image set A are depicted for the following: (**A**) one of the images where AxonJ performance was within 95% of the expert count, and (**B**) an image where AxonJ performance had only 88% correspondence to the expert.

We developed the AxonJ algorithm (Fig. 1) through re-iterative comparisons of automated and manual counts of PPD-stained axons (Fig. 2). The AxonJ axon counting and axon density algorithm performs the following well-established image analysis steps on an input image, as illustrated in the flowchart in Fig. 1:

#### a. Local Histogram Equalization

Local histogram equalization is performed to enhance image contrast^20^, with a block-size of 63 pixels. 256 histogram bins, and a maximum slope of 3.

#### b. Axonal Membrane Segmentation

The axonal membrane is then segmented by first applying a ridge detector kernel, implemented as the largest eigenvalue of the Hessian matrix-the local second order derivative matrix-as follows^21^.


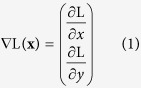



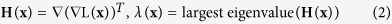


Where *L(***x**) is the image, ∇L(**x**) is the image gradient at **x**, H(**x**) is the Hessian matrix at **x**^22^. A σ of 1.6, and 4.0 were used for the 40x, and 100x images respectively. A mean threshold is then used to create a binary image of the axonal membranes. The mean threshold, after local histogram equalization, is determined dynamically and automatically for each image, and does not require user input.

#### c. Counting Connected Regions

Connected regions are identified as described by Niemeijer *et al.*^22^, and then extracted as axon candidates. Axon candidates are rejected if they are larger than 14.4 μm^2^ (5000 pixels at 40x magnification) or smaller than 0.144 μm^2^ (50 pixels at 40x magnification) (Fig. 1). These size thresholds were previously determined empirically on a separate set of images to balance competing concerns. The lower threshold is a compromise between excluding the real small-sized axons and over counting connected regions that are not axons, as lower threshold values rapidly add spurious elements. The upper threshold is a compromise between excluding large-sized axons and glial masses, as higher threshold values resulted in larger glial cells and masses being counted in the axon count measures. Thus, filtering of ambiguous and invalid regions is performed, and axonal candidates are rejected if they are outside this calibrated axon size distribution. In our preliminary studies, we observed that it is not appropriate to exclude axonal candidates based on circularity, as there are expert segmented axons that exhibit circularity measures across the entire range. Many digital image backs for microscopes store the image resolution in the image file. The size thresholds and the scale for the Hessian convolution^21^ are therefore scaled appropriately based on image resolution automatically if available. If the image resolution was not stored or is otherwise not available, AxonJ requests the user to input the resolution of the image in pixels/μm manually (as known distances in pixels and micrometer).

Thus, AxonJ is designed to be robust across varying scales – as long as axons are at least several pixels in size, they will be counted. Because of the robustness of the filters that AxonJ uses, we have not found a sensitivity to average image intensity (exposure) in the variety of image sets we have tested.

AxonJ excludes glial masses based on size, dying (dark) axons because they are heavily pigmented throughout, and cells that are not axons because their size is larger than the allowed axon size range. Abnormally shaped axons are counted by AxonJ as axons.

The algorithms were implemented in Java as AxonJ, an ImageJ plugin available in the ImageJ plugin repository at [imagej.nih.gov/ij/plugins/axonj/]. AxonJ takes approximately 10 minutes on a standard desktop PC to count all axons in an optic nerve section.

### Comparison of Optic Nerve Section Quantification by AxonJ and Human Experts

**Figure 3:**
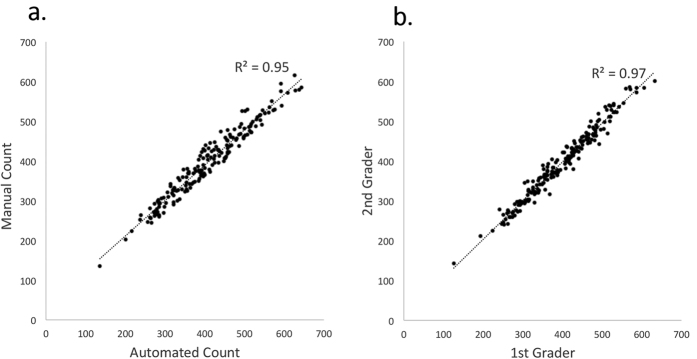
AxonJ counts and manual counts at 100x for image set A. (**a**) The reference standard with the average of manual axon counts by two experts on the Y-axis and AxonJ counts on the X-axis; (**b**) the counts of the two experts on the X- respectively Y-axis.

We tested AxonJ’s axon counting performance with a battery of validation assays that compare AxonJ to human experts in multiple ways (Table 1). We evaluated AxonJ by comparing its total optic nerve axon counts with counts produced by human experts. Sections of optic nerves were prepared and axons were labeled with PPD, as previously described (see Methods). Images of the optic nerves were then captured using a bright-field microscope with a 100X objective lens. We first compared the total axon counts manually obtained by each of the two masked experts using optic nerve sections from a colony of mice on the C57BL/6J genetic background (n = 19, image set A in Table 1). Ten images representing 10% of the total area of the optic nerve section were analyzed from each sample. With this approach, the experts produced axon counts that are highly concordant with each other (r^2^ = 0.97, 95% CI 0.962–0.978). The average of the axon counts made by the two experts was considered the reference standard. Next, axon counts were obtained from the same optic nerve sections using AxonJ and were compared with the reference standard. A high level of concordance was detected between AxonJ counts and the reference standard (r^2^ = 0.95, 95% CI 0.936–0.964) validating the accuracy of the algorithm (Fig. 3).

Next, the cross-sectional area of the same optic nerves was measured and axon density computed. As expected, the highly correlated manual axon counts from the two experts produced axon density measurements that were also highly correlated (r^2^ = 0.97, 95% CI 0.962–0.9783). Axon density was also calculated using axon counts by AxonJ. The AxonJ algorithm-generated axon density was highly correlated with density calculated using the averaged manual axon counts from the experts, r^2^ = 0.95 (95% CI 0.936–0.964).

Using the average of the expert axon counts as a reference-standard, we assessed the accuracy of automated axon counting by AxonJ (see Online Methods) in image set A. AxonJ had a sensitivity of 0.96 and overall precision of 0.91, determined from the 100x images (Table 1 and Supplemental Table 2) with 65,498 true positive axons, 6,706 false positive axons, and 2,738 false negatives, see Fig. 3.

The above experiments were performed on images captured with a 100x objective, a standard magnification used in most published studies of optic nerve axon counts. One disadvantage of using images obtained with a 100X objective is the small field of view, which requires numerous images to completely cover an optic nerve with overlapping images. Consequently, we investigated the effect of using fewer lower-magnification images that span the entire optic nerve. We re-analyzed the same optic nerves using images obtained with a 40X objective. These images have a much broader field of view and only 4 to 6 are needed to cover an entire optic nerve section. Images from optic nerve sections labeled with PPD were obtained from the same optic nerve preparations (n = 19, image set A in Table 1) using the 40X objective lens.

Registered montages were created by fusing the 4–6 images spanning each optic nerve, and AxonJ was used to count the total number of axons in each of these whole nerve sections. These total optic nerve axon counts made with the 40X lens were then compared with total axon counts extrapolated from the counts made sampling 10% of the optic nerve with the 100X lens as described above. The axon counts made by AxonJ using the 40X objective closely corresponded with the reference standard made at 100X, r^2^ = 0.87 (95% CI 0.679–0.947). The axon count data is listed in Table 1 and Supplemental Table 2. Because the images collected with a 40x objective yielded highly repeatable data comparable to those collected with a 100x objective, and gave the significant advantage of being able to readily quantify entire optic nerve cross sections, we subsequently performed all analyses using 40x.

We evaluated the precision of the AxonJ algorithm by comparing axon counts from serial sections histologically cut from the same tissue blocks (n = 20 optic nerves, image set B, see Table 1) and imaged with a 40x objective. Because the sections are from an individual nerve, but likely to contain slightly different sectioning and staining artefacts, this experiment represents a stringent test of repeatability. AxonJ serial section repeatability was high, with two serial sections of the same optic nerve giving an r^2^ = 0.95 (95% CI 0.936–0.964) (Supplemental Table 2). Together, these results indicate that AxonJ is capable of quantifying axon number similarly to human experts.

**Figure 4:**
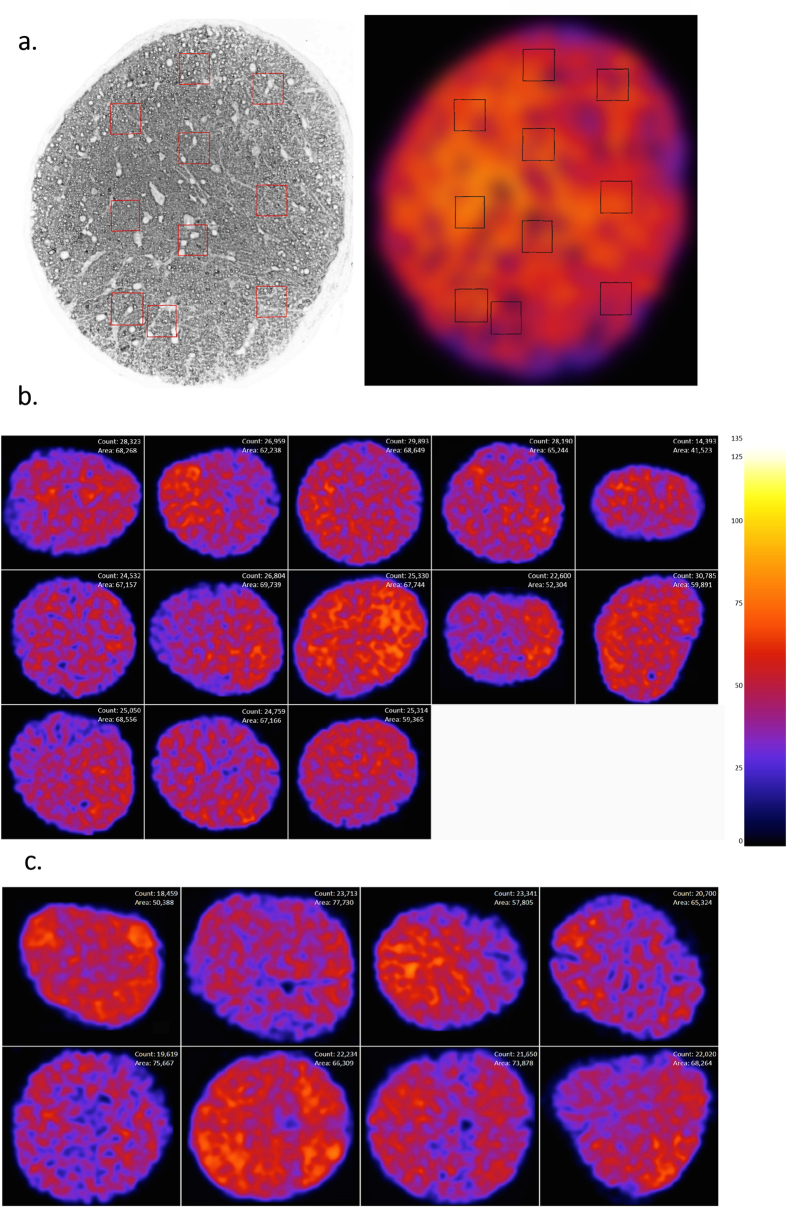
AxonJ axon density heat maps. (**a**) AxonJ generated heat map of a whole optic nerve section showing the subimages that were sampled for manual counting by human experts indicated by boxes. (**b**) Density heat map for strain matched *wt* mice (image set C0), (**c**) density heat map for *nee* mice (image set C1), demonstrating the regional variation in density as well as optic nerve cross-sectional area. The heat map shows denser areas of axons as hotter, while colder regions represent less dense regions of axons. Whole-nerve axon count and optic nerve area (μm^2^) are provided for each cross section. Scale bar (axons/100 μm^2^).

Most optic nerve studies with mice have focused on axon number, with relatively few studies commenting on axon density throughout the entire nerve cross section^23^. Using AxonJ axon counts, we calculated the coefficient of variation of axon counts for the different strains, see Table 1. From our analyses with AxonJ, we also observe that axon density is not homogeneous across the nerve and axon density distributions differ substantially between individual mice (Fig. 4).

To illustrate the detection of axons by both experts and AxonJ, we show PPD-stained 100x optic nerve sections from image set A (Table 1) in Fig. 2, with as the axons identified by the experts marked with red points and the number 7), and the axons identified by AxonJ outline outlined in light blue. 157/190 of the images were within 95% of the expert count, and one of these is show in Fig. 2A, while 33/190 of the images had less than 95% correspondence with the expert count, and one of these is shown in Fig. 2B.

### Robustness to image resolution

As described above, we use filters that are robust to image scale (i.e. the size of axons in pixels), to test this we determined the AxonJ count after downsampling an optic nerve section (Image Set A, Table 1) across a range of resolutions, showing that we can accurately measure the number of axons using AxonJ across resolutions. The relationship between axons counted is relatively flat (Supplemental Fig. 1). The manual count (on full resolution only, we have not determined manual counts on different resolutions) is indicated with a red line for comparison. The small differences are most likely caused by the fact that the smallest axons become less than the few pixels needed to distinguish them from the background. Thus, the lower the resolution, the more small axons are lost. These images at different resolutions are available in the AxonJ plugin distribution from the ImageJ website.

### Validation of AxonJ on previously published datasets

**Figure 5:**
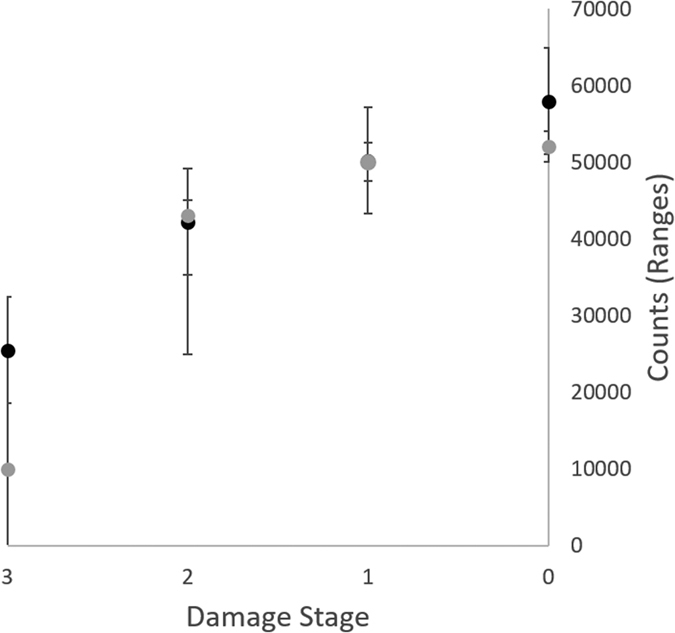
Comparison of AxonJ whole nerve counts to previously published glaucoma damage staging on the same DBA/2J, image set E. Average of AxonJ axon counts (in black) and the expert-determined average axon counts (in gray) for each optic nerve damage staging (normal and damage stages 1–3).

DBA/2J are a widely used strain of mice for glaucoma research. Previous studies established a 3 stage glaucoma damage grading system (normal and increasing damage stages: 1-mild, 2-moderate, and 3-severe)^7^,^9^,^24^ to determine the presence and severity of glaucoma in these mice. Original DBA/2J optic nerve sections from a previously published study in which the same samples had already been graded were kindly made available to us for reanalysis with AxonJ^25^. As a rigorous test of AxonJ performance, we quantified the axons in these sections at 40x (image set E, n = 20, Table 1 and Supplemental Table 2) and compared axon counts and density to the original glaucoma damage stages. AxonJ-derived axon number was tightly correlated to this previously published glaucoma damage staging (r_s_ = 0.882, P = 4.9 × 10^−5^), see Fig. 5^26^. AxonJ-derived axon density was even more tightly correlated with prior glaucoma damage staging (r_s_ = 0.905, P = 6.4 × 10^−8^).

Similar comparisons were also made using previously published data on *nee* mice, a strain with congenital defects leading to an early-onset form of glaucoma. Previous studies established axon counts at P17 and P90 to determine the presence and severity of glaucomatous damage in mice homozygous for the *nee* mutation versus littermate controls without the mutation (*wt*)^27^. The original optic nerve sections used were available for re-analysis with AxonJ. We quantified the axons in these sections using AxonJ and determined whether AxonJ could measure a difference between *nee* and *wt* at both P17 and P90. At P17, an age at which axon myelination is not yet complete in *nee* mice, average axon count (±SD) in *nee* mice (set C1) was 21,840 ± 2,473 and for strain-matched *wt* (set C0) was 25,575 ± 4,138, a significant difference (p = 0.018). At P90, when most *nee* optic nerve axons have degenerated, average axon count in *nee* mice (set D1) was 24,998 ± 2,902, and for strain-matched *wt* (set D0) was 40,658 ± 10,250, also a significant difference (p = 0.028). At P17, correlation between previously published counts^27^ and AxonJ counts was r^2^ = 0.70, (95% CI 0.633, 0.931), and at P90, when most *nee* optic nerve axons have degenerated, correlation was r^2^ = 0.75 (95% CI 0.661, 0.95). Thus, automated analysis with AxonJ again recapitulated the main findings of a previously published dataset originally studied by manual methodology.

## Discussion

We have presented AxonJ, a fully automated axon counting tool that allows all axons stained with PPD in rodent optic nerve sections to be counted. Our results show that AxonJ performs comparably to human experts on optic nerve sections imaged with a standard light microscope with a 40x objective lens. Because of the large increase in efficiency, i.e. counting all axons in minutes, AxonJ allows all axons in the entire nerve to be counted as well as axon size distribution and axon density over the entire nerve to be quantified.

The parameters of AxonJ were optimized on a small set of optic nerve sections, distinct from those used in the present study. These parameters were the scale of the Hessian convolution and the minimal and maximal cross-sectional area limits. Since that initial optimization, these parameters have remained unchanged, except for requisite re-scaling as the resolution (pixels/μm) of the image requires.

AxonJ was validated at the sample level by comparing automated axon counts to standard manual counts by two masked, independent experts on small areas of the optic nerve section at 100x. The results show that AxonJ counts are comparable to the average human expert axon counts from those samples. This conclusion was confirmed by comparing full counts of the whole optic nerve section using 40x magnification images of the whole nerve section against extrapolated whole nerve counts on the basis of the 100x samples (image sets A and C, Table 1, Fig. 3), and also by the high serial-section reliability, where we compared full counts of two consecutive sections from the same optic nerves (image sets B, Table 1). AxonJ sensitivity and precision were both high. In a sample-level analysis, AxonJ counts generally exceeded manual counts. This pattern can best be attributed to imperfections in the segmentation step – more advanced segmentation methods can be utilized to improve the algorithm and exclude noise particles. Importantly, our results were also confirmed on a previously published dataset, using optic nerve section images from DBA/2J mice with a late-onset form of glaucoma^7^,^9^,^24^,^25^. AxonJ counts and axon density measurements, when applied to the DBA/2J images in a masked fashion, corresponded well to the glaucoma damage staging, in fact the correlation of AxonJ axon density to glaucoma damage staging was remarkably strong at r_s_ = 0.905. (Fig. 5). While the current iteration of AxonJ produces highly accurate counts of axons in normal to moderately damaged optic nerves, its performance in severely damaged optic nerves is adequate but less strong, confirming its utility in studying mouse models of glaucoma.

Many currently used axon counting methods rely on sampling a subset of the optic nerve as a means of estimating total number of axons, such as random and targeted sampling counting strategies^28^,^29^,^30^. Thus, a central assumption of these methods is that axon density is homogeneous across the nerve^23^. Our results (Fig. 4) show that this assumption may not be valid in mice. We found large regional variation in axon density across optic nerve sections, as well as variations between optic nerves of individual mice. The coefficient of variation for axon number in adult C57BL/6J optic nerves has previously been reported to range from 3.6%–8.9%^8^,^10^,^11^, lower than the 12.4–19.8% we saw in adult mice, and also lower than the 11.3%–16.2% we saw in P17 mice (Table 1). Because we and others^13^ have observed that the cross sectional area of the optic nerve is variable in mice, axon density may thus be a more uniform metric than the absolute number of axons; additional studies of this possibility are warranted.

AxonJ is publicly available in the ImageJ plugin repository at [imagej.nih.gov/ij/plugins/axonj/]. As any ImageJ plugin, AxonJ can be built upon by other groups for more complex applications including interaction with microscopy stages.

AxonJ is thus of value for the study of optic neuropathies such as glaucoma, but also for other diseases that affect retinal ganglion cells, such as diabetes (Sohn *et al.*, PNAS in press 2015) and the mechanics of genes that influence retinal ganglion cell number. Another advantage is that repeat counting of the same section with AxonJ yields an essentially identical count and density, so that two labs analyzing the same dataset will get the same answer. Finally, automated microscope and stage set ups have become available and allow whole nerve cross-sections to be imaged at high magnification and counted manually^30^. However, the huge resources required to manually count all nerves, make these systems expensive in practice. Obviously, coupling such automated microscopy platforms with computational axon analysis algorithms such as AxonJ, will improve the cost-benefit ratio, enabling whole-nerve axon counting as a common practice with the ability to capture and quantify small axons^31^. Though this would be an advantage, AxonJ does not rely on any hardware that is not present in a standard lab, nor does it require any specialized husbandry such as to tagged transgenics.

There are some caveats to our implementation of AxonJ and opportunities for improvement. First, the use of 40x magnification images limits the resolvability of some axons. It is likely that axons of smaller diameter may go undetected and thus may limit the accuracy of using 40x images for AxonJ to count all axons. However, the smallest axons cannot be imaged at all with light microscopy, so this is essentially a limitation of all light microscopy based methods^23^. Second, AxonJ relies on the high contrast resulting from PPD labeling of the myelin sheath of optic nerve axons. Thus, unmyelinated axons go undetected and may reduce the accuracy of AxonJ counts. Third, while assessing circularity of the optic nerve is a reasonable test to insure that histologic cross sections were not cut obliquely across the entire nerve, some nerves are likely to have compressed or oblique sub-areas within them that cannot be analyzed with AxonJ. Finally, the results show that the current version AxonJ is optimal for healthy mice and glaucomatous mice with glaucoma damage staging 1–2, while in more severe damage grades (i.e. stage 3), AxonJ counts slightly overestimate. This is likely because with substantial axon loss, it becomes harder to distinguish between axonal and non-axonal objects, including glial structures. In addition, a general limitation of any axon counting method is that it may not accurately reflect the number of surviving RGCs, so some studies will still benefit from analyzing both the nerve and retina.

The focus of AxonJ is automated counting, and its goal is increasing lab productivity and objectivity. Adding manual correction capabilities would eliminate the automation of axon counting by AxonJ and would require hours and even days of extra analysis. Though it might be possible to add editing capabilities for AxonJ, we felt this is beyond the scope of the present study. Instead, our approach has been to filter out those candidate axons that are too large, such glial masses, or that are heavily pigmented throughout, such as dying axons.

AxonJ counts axons in light microscopy images. We are not aware of any automated axon counting algorithms for electron microscopy (EM) images^10^,^28^,^29^,^30^,^32^,^33^,^34^,^35^,^36^,^37^. Semi-automated counting methods of axons in transmission EM images have been published^36^,^37^. These are sampling based methods that count individual patches, instead of the whole optic nerve as does AxonJ, and lack validation on external published datasets^36^.

In summary, AxonJ, is a fully automated axon counting tool that allows axons stained with PPD in rodent whole optic nerve sections to be reliably quantitated. Our results show that it performs comparably to human experts on 100x samples of optic nerve sections from mice on the C57BL/6J genetic background, with r^2^ = 0.95, high inter-observer reliability of r^2^ = 0.97, high serial section repeatability r^2^ = 0.95, and high correspondence with glaucoma damage staging^7^. Because of the increase in efficiency, AxonJ allows all axons in an entire nerve section, as well as axon density, to be quantified in minutes. It is publicly available, only requires standard tools and hardware, and has the potential to improve axon assessments in rodent models of disease method for a better understanding of eye diseases, and eventually preventing visual loss and blindness.

## Online Methods

### Mice

All Iowa animals were treated in accordance with the ARVO Statement for the Use of Animals in Ophthalmic and Vision Research. All experimental protocols were approved by the Animal Care and Use Committee of the University of Iowa.

One large colony of mice was previously generated in collaboration with the Transgenic Animal Facility at the University of Iowa by injecting a bacterial artificial chromosome (RP11-60O8, BACPAC, Oakland, CA) containing the wild-type human *TBK1* gene (natural promoter, exons, and introns) into C57BL/6J/SJL hybrid embryos. A founder was backcrossed to C57BL/6J mice for 4–5 generations at the onset of this study and >10 generations by conclusion of the study). The integration of a single BAC was determined using a commercially available real-time PCR assay (TaqMan, Life Technologies, Grand Island, NY) on all mice. Hemizygous mice (B6-Tg(*RP11-6008*)1Fin^+/Tg^) and littermate non-transgenics (B6-Tg(*RP11-6008*)1Fin^+/+^) were both included in the study for dataset A. For the validation of AxonJ using previously published datasets, *nee* refers to B10.A-H2h4/(4R)SgDvEgJ mice homozygous for the *nee* mutation in the *Sh3pxd2b* gene^27^ (dataset C and D, see Table 1), and DBA/2J refers to standard inbred mice with mutations in the *Tyrp1* and *Gpnmb* genes^38^ (dataset E). Controls for *nee* mice were littermates homozygous for the wild-type *Sh3pxd2b* allele from the *nee* colony. Tissue sections used with the *nee* model are identical to sections presented in of Mao *et al.*^27^; tissue sections used with the DBA/2J model are identical to sections presented in Howell *et al.*^35^.

### Optic Nerve Image Preparation

Standard techniques were used to prepare and image optic nerve cross-sections. After sacrificing the mouse, the head and eyes were removed. The head and brain were fixed in a 1/2 K buffer overnight. The optic nerves were dissected from underneath the brain and removed the next day, followed by further fixation, dehydration by ethanol, and embedding in resin over a three day period. The entire process takes five days. 0.5 μm sections were acquired using a Leica EM UC5 Ultramicrotome with a Histo Diamond Knife. They were stained with a 1% paraphenylenediamine (PPD) in a 1:1 isopropanol:methanol solution for one hour, rinsed twice with 1:1 isopropanol:methanol for five minutes, and finally rinsed with xylenes for three minutes.

### Optic nerve Imaging

The following standard pre-processing steps were applied to all sections. The Olympus BX52 microscope with an Olympus DP72 camera back was used to image the optic nerve cross-sections using manual adjustments with 40x and 100x objectives, at 4140 × 3096 pixels. This resulted in a measured 40x objective resolution of 18.65 pixels/μm, and 100x objective resolution of 45.45 pixels/μm, we use 40x and 100x to indicate the images thus obtained respectively (Table 1). At 40x, imaging yields four to six raw images with varying amounts of overlap to cover the whole nerve. 40x was selected as the empirically optimal level of magnification for imaging. At 100x, imaging yielded 10 images per nerve, with areas randomly sampled from the whole nerve, so these images contain no overlap. The total area of the optic nerve cross-sections were determined by manually outlining the optic nerve border in ImageJ. The measured nerve area was used to determine the size of the counting frame for manual counting. A breakdown of the different datasets prepared and analyzed in this study is provided in Table 1, giving mouse model, number of mice, magnification, and number of images for each magnification.

### Manual Counting of Axons

To manually count axons, a counting frame was placed on the ten 100x images, and myelinated axons, within the frame and intersecting the upper and left edges, were marked and counted manually using ImageJ according to standard unbiased counting rules^39^. The same-sized counting frame was used on all ten images sampled from a single mouse. However, the size of the counting frame varied across different mice due to variations in optic nerve area. A single counting frame was calculated to equate to 1% of the total optic nerve area so that a total of 10% of the optic nerve cross-section was counted for each mouse. The identity of images was masked before counting and analysis. Manual axon counts were obtained by two masked, trained human observers (KM and AT) from cropped 100x optic nerve images and then averaged for each nerve as the per image reference standard. A per whole nerve reference standard was obtained by sampling 10% of the section and averaging the counts at 100x by these two masked experts^27^, followed by extrapolation of the counts to the entire section surface. One expert marked all axon centroid locations that expert counted, so that we had axon locations for all images, used as the *detection* reference standard to determine sensitivity and precision.

### Optic Nerve Measurements

Optic nerve cross-sectional area and circularity were determined by manually tracing the optic nerves using ImageJ. Axon density (axons/μm^2^), was calculated by normalizing the software-generated whole nerve axon counts by the measured cross-sectional area. Circularity of the optic nerve was defined and determined by ImageJ’s circularity metric^18^.


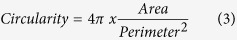


where, *Area* and *Perimeter* are the area and circumference respectively of the optic nerve cross-section.

### Using AxonJ

For 100x images, no preprocessing was performed, as these were analyzed individually. For whole optic nerve section images obtained at 40x, an image registration step is first performed to obtain a single merged image for the entire section, using standard methods^20^,^40^,^41^. AxonJ is then applied, as explained in the Results section.

### Optic Nerve Section Quantification with AxonJ

Comparisons of AxonJ and corresponding reference standard axon counts and axon densities were compared by Pearson’s correlation coefficient r^2^, if counts were available, and calculated as -r_s_ using Spearman’s Rank-Order Correlation where glaucoma damage stagings were available for each nerve^42^ AxonJ Serial-Section Repeatability was calculated from AxonJ counts on two consecutive, serial sections of the same optic nerve (each 0.5 μm thick) at 40x at 18 months, in Tg-(RP11-6008)1Fin^+/Tg^ and strain-matched *wt*, Tg-(RP11-6008)1Fin^+/+^, using Pearson’s correlation coefficient r^2^. AxonJ Sensitivity and Precision was calculated using the detection reference standard determined by one expert, and the boundary of each axon detected by AxonJ. True positives were defined as AxonJ determined axon boundaries that contain reference standard axon detections. False positives were defined as AxonJ determined boundaries that did not contain a reference standard axon detection. False negatives were defined as a reference standard detection not contained within any AxonJ determined boundary. Sensitivity and Precision were then calculated using the standard equation


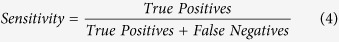



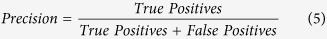


Axon-level assessment of true negatives was not possible, so specificity and accuracy could not be computed. Axon density heat maps were constructed for the 40x *nee* and *wt whole* optic nerve images (image sets C, n = 21, Table 1 and Supplemental Table 1), by counted the axons in a moving window of 100 × 100 pixels (28.75 μm^2^), and color mapping encode the regional axon density at each pixel in each whole nerve image.

## Additional Information

**How to cite this article**: Zarei, K. *et al.* Automated Axon Counting in Rodent Optic Nerve Sections with AxonJ. *Sci. Rep.* **6**, 26559; doi: 10.1038/srep26559 (2016).

## Supplementary Material

Supplementary Information

## Figures and Tables

**Table 1:** Mouse Models/Experiment/Dataset Legend.

Dataset (as in text)	Genotype	Timepoint Sacrificed	n (mice)	Magnifications used	N40 (number of registered 400x whole nerves)	N100 (number of 100x images)	Coefficient of Variation of AxonJ counts
A	B6-Tg(*RP11-6008*)1Fin^+/+^ and B6-Tg(*RP11-6008*)1Fin^Tg/+^	7 mo, 12 mo	19	40x, 100x	19	190	13.1%
B	B6-Tg(*RP11-6008*)1Fin^+/+^ and B6-Tg(*RP11-6008*)1Fin^Tg/^	18 mo	20	40x	20	N/A	19.2%
C0	*B10-Sh3pxd2b*^*+/+*^	17 days	13	40x	13	N/A	16.2%
C1	*B10-Sh3pxd2b*^*nee/nee*^	17 days	8	40x	8	N/A	11.3%
D0	*B10-Sh3pxd2b*^*+/+*^	90 days	5	40X	5	N/A	25.2%
D1	*B10-Sh3pxd2b*^*nee/nee*^	90 days	12	40x	12	N/A	11.6%
E	*DBA/2J*^7^,^26^,^35^	N/A-Varying damage stages	20	40x	20	N/A	
