# Corrigendum: Automated Axon Counting in Rodent Optic Nerve Sections with AxonJ

**DOI:** 10.1038/srep34124

**Published:** 2016-10-19

**Authors:** Kasra Zarei, Todd E. Scheetz, Mark Christopher, Kathy Miller, Adam Hedberg-Buenz, Anamika Tandon, Michael G. Anderson, John H. Fingert, Michael David Abràmoff

Scientific Reports 6: Article number: 2655910.1038/srep26559; published online: 05 26 2016; updated: 10 19 2015

In the Supplementary Information file originally published with this Article, Supplemental Figure 1 was omitted. In addition,

“Supplemental Software”

was incorrectly given as

“Software Legend”

These errors have now been corrected in the Supplementary Information that now accompanies the Article.

