# Supplementary material for: Automated Axon Counting in Rodent Optic Nerve Sections with AxonJ
Source: Sci Rep. 2016 May 26;6:26559. doi: 10.1038/srep26559 (PMC4881014; doi:10.1038/srep26559)
Supplement: Supplementary Information [file srep26559-s1.pdf]

Supplemental Information

SREP-15-32954A R3

Automated Axon Counting in Rodent Optic Nerve Sections with AxonJ

Kasra Zarei,[1,2,] Todd E. Scheetz,[1,2,3] Mark Christopher[1,2,], Kathy Miller,[1,3] Adam Hedberg-Buenz,[1,5, 6] Anamika Tandon,[1,3] Michael G. Anderson,[1,3,5, 6] , John H. Fingert,[1,3] Michael D. Abràmoff[1,2,3,4,5]

Supplemental Table 2: Validation experiments

Experiment	Image sets used (see Table 1)	n (mice) (single eye per mouse)	n (40x) (whole nerve section images)	N (100x) images)	r^2	p-value
AxonJ counts vs. average of two expert's counts	A	19	-	190	0.95	3.01×10^{-124}
100x Image Samples - expert 1 counts vs. expert 2 counts	A	19	-	190	0.97	4.17×10^{-145}
Whole nerve AxonJ counts (at 40x) vs. extrapolated expert counts (at 100x)	A	19	19	190	0.87	1.0×10^{-8}
Whole nerve AxonJ counts (at 40x) vs. extrapolated published expert counts (at 100x)	C0, C1	21	21	-	0.70	2.36×10^{-6}
Whole nerve AxonJ counts (at 40x) vs. published	D0, D1	17	17	-	0.75	7.02×10^{-6}

extrapolated expert counts						
AxonJ Serial-section Repeatability - AxonJ count of section 1 vs. AxonJ count of next section 2,	B	20	20	-	0.95	5.89×10^{-13}
AxonJ DBA/2J Axon Counts vs. Glaucoma Damage staging	E	20	20	-	ρ = -0.807	4.9×10^{-5}
AxonJ DBA/2J Axon Density vs. Glaucoma Damage staging	E	20	20	-	ρ = -0.888	6.4×10^{-8}

Supplemental Table 3: Characterization and Comparison of Metrics for *nee* and strain matched *wt* mice

**Genotype**	***nee* (N1, n = 8)**	***wt* (N0, n = 13)**	**P-value**
Whole optic nerve (40x) counts	21,840 ± 2,473	25,575 ± 4,138	0.018
Area (μm^2)	66,921 ± 9,281	62,911 ± 8,150	0.333
Axon Density (axons/ μm^2)	0.330 ± 0.046	0.407 ± 0.047	2.5 x 10^{-3}

Supplemental Figure Legend

Supplemental Figure 1: Comparison of AxonJ counts and image resolution - the following figure shows, by showing the AxonJ determined count after down-sampling the image (Image Set A, Table 1) across a range of (equivalent) resolutions, that AxonJ accurately measure the number of axons. The relationship between axons counted and image resolution is relatively flat. The manual count (on full resolution only) is indicated with a red line for comparison. The small differences are most likely caused by the fact that the smallest axons become less than the few pixels needed to distinguish them from the background. Thus, the lower the resolution, the more small axons are lost.

Software Legend

AxonJ is available at http://rsb.info.nih.gov/ij/plugins/axonj/index.html. The software package contains the AxonJ plugin, three sample image patches with the acquired image resolutions saved in the TIFF images, and an HTML file containing installation details about AxonJ.
